# Further validation of the affective bias test for predicting antidepressant and pro-depressant risk: effects of pharmacological and social manipulations in male and female rats

**DOI:** 10.1007/s00213-017-4687-5

**Published:** 2017-07-22

**Authors:** Justyna K. Hinchcliffe, Sarah A. Stuart, Michael Mendl, Emma S. J. Robinson

**Affiliations:** 1 School of Physiology, Pharmacology and Neuroscience, University of Bristol, Biomedical Sciences Building, University Walk, Bristol, BS8 1TD UK; 2 School of Veterinary Sciences, University of Bristol, Langford House, Langford, Bristol, BS40 5DU UK

**Keywords:** Major depressive disorder, Antidepressants, Stress, Corticosterone, Enrichment, Affective bias

## Abstract

**Rationale:**

Affective biases are hypothesised to contribute to the cause and treatment of mood disorders. We have previously found that affective biases, associated with learning and memory, are observed following acute treatments with a range of antidepressant and pro-depressant manipulations.

**Objective:**

This study aimed to test if similar biases are observed in male and female Sprague Dawley (SD) rats. We also test whether the stress hormone, corticosterone, induces a negative bias in the affective bias test (ABT) consistent with its putative role in the development of depression. We then use a meta-analysis to compare our findings with data published for the Lister Hooded rats.

**Methods:**

The ABT uses a within-subject study design where animals learn to associate distinct digging substrates, encountered on different days, with the same value food reward. Exposure to one substrate is paired with a treatment manipulation (drug or environmental) and the other with a control condition. A preference test is used to test if the treatment has induced a positive or negative bias.

**Results:**

Consistent with previous data, both male and female SD rats exhibit similar positive affective biases following treatment with the antidepressant, venlafaxine, and social play and negative affective biases following FG 7142 (benzodiazepine inverse agonist) and social stress. Acute treatment with corticosterone induced a negative bias.

**Conclusions:**

These data add to the translational validity of the ABT and suggest that corticosterone can induce a negative affective bias following acute treatment, an effect which may contribute to its long-term effects on mood.

## Introduction

Mood disorders, such as major depressive disorder (MDD), affect approximately 10–15% of the population and, in the future, will be one of the most prevalent disorders, severely affecting quality of life (Beck 2008; Disner et al. 2011). There is a wide spectrum of symptoms associated with MDD, including physiological (e.g. loss of energy, sleep disturbances) and psychological (e.g. low mood, anhedonia, cognitive impairments, suicidal thoughts), adding complexity to the study of the underlying pathophysiology (Clark et al. 2009; Roiser et al. 2012).

Emotional states play a crucial role in perception, selection and modulation of information processing (Clark et al. 2009; Roiser et al. 2012). Human studies have shown that MDD significantly influences affective processing leading to a range of cognitive changes (Clark et al. 2009; Roiser et al. 2012). Depressed patients show negative biases in emotional recognition and interpretation, pessimistic interpretation of information, negative autobiographical memory and exaggerated reactions to negative feedback (Clark et al. 2009; Harmer et al. 2009a; Harmer et al. 2009b; Roiser et al. 2012). These cognitive impairments are thought to contribute to the persistence of mood disorders (Beck 1970, 1976, 2008; Disner et al. 2011). Recent studies in humans and animals have also suggested that reversal of these affective biases may be an important neuropsychological mechanism for antidepressant therapy (Hales et al. 2014; Harmer et al. 2009a; Stuart et al. 2013, 2015). Patient and healthy volunteer studies suggest that antidepressants can induce positive biases following acute administration without affecting subjective reporting of mood. It is therefore suggested that the delayed onset of action of antidepressants may involve neuropsychological mechanisms via which these objective changes in affective bias reverse the patient’s negative biases until they become subjectively aware of their improved affective state (see Harmer et al. 2017, 2009a for a more detailed discussion).

The use of animal paradigms with high predictive and translational validity enables a better understanding of the underlying pathophysiology of MDD; however, the models and tests used for depression research have been widely criticised (Berton and Nestler 2006; Cryan and Holmes 2005; Nestler and Hyman 2010). The concept of affective biases in animals has developed since the first study published by Harding et al. (2004) provided evidence supporting cognitive biases in non-human species (also see Hales et al. 2014 for a review). From this initial work, two main types of affective bias task have developed: the judgement bias task (JBT), which measures interpretation biases (Enkel et al. 2010; Harding et al. 2004; Papciak and Rygula 2017; Rygula et al. 2013; also see Hales et al. 2014 for a review), and the affective bias test (ABT), which measures biases associated with learning and memory (Stuart et al. 2013, 2015).

In the present study, we investigated affective biases associated with learning and memory using the ABT in both male and female Sprague Dawley (SD) rats, a strain commonly used for MDD research. We tested whether previous findings observed in Lister Hooded (LH) rats (Stuart et al. 2013, 2015) could be replicated in a different strain of rat, the albino SD. Investigation of generalisation across strains is important given that previous studies have revealed strain-associated differences between SD, Wistar, Fischer F344 or Long Evans rats in learning processes, in drug- or stress-induced responses, toxicology, endocrinology and enzymatic system (see review of Kacew and Festing 1996). Several studies have shown increased sensitivity to putative negative manipulations in SD rats, and comparison of hypothalamus–pituitary–adrenal axis (HPA) activity in adult male rats during standard laboratory procedures, e.g. handling for the first time, showed that corticosterone concentration was higher in SD than in Lewis rats (Deutsch-Feldman et al. 2015). Our previous work has only used male rats, and so we also included a separate study in female SD rats to investigate if there were any sex differences in the manipulations tested in the ABT. Previous studies using male versus female rodents have found differences in their responses in various paradigms and are not always consistent with the clinical picture where females appear to be more vulnerable to mood disorders (Dalla et al. 2010; Joel and Yankelevitch-Yahav 2014). We then used a meta-analysis to compare the male versus female SD rats with our previous published work from male Lister Hooded rats.

Based on previous results (Stuart et al. 2013, 2015), we tested two different pharmacological compounds: the serotonin–noradrenaline reuptake inhibitor, venlafaxine and the anxiogenic benzodiazepine inverse agonist, FG 7142. We also tested both positive and negative psychosocial manipulations of affective state using social play and restraint stress and social isolation, respectively. As corticosterone has been associated with causing depression-like effects following chronic administration (Gregus et al. 2005; Ulloa et al. 2010), we also tested whether acute treatment would induce a negative bias in the ABT.

## Methods

### Animals and housing

Subjects were 12 male and 12 female SD rats (Charles River, UK). Male rats weighed approximately 300–350 g and females weighed 200–250 g at the start of experimental manipulations. The rats were housed in same-sex pairs in enriched laboratory cages (55 × 35 × 21 cm) with sawdust, paper bedding, red Perspex houses (30 × 17 × 10 cm), cotton rope and cardboard tubes in temperature-controlled conditions (21 ± 1 °C) and under a 12:12-h reverse light–dark cycle (lights off at 07:00 h). Rats were mildly food restricted to approximately 90% of their free-feeding weights (~18 g of food per rat/day laboratory chow (Purina, UK)). Water was freely available, except during the pairing and test sessions. The behavioural procedures and testing were performed during the animals’ active phase between 09:00 and 17:00 h.

### Apparatus

The ABT was conducted in a Perspex arena (40 × 40 cm) with the digging substrates placed in two glazed pottery bowls (*Ø* 10 cm). The substrates were matched for similar digging effort, and new sets of substrates (A or B (reward-paired substrates) versus blank (unrewarded substrate used in the pairing sessions); see Table 1 for details) were used for each testing week of the experiment. The digging substrates were placed in bowls and presented in a pseudo-random order in the left or right position within the arena.

**Table 1:** List of the substrates used in the experiments

	Substrate ‘A’	Substrate ‘B’	Substrate ‘blank’
Test 1	Felt	Shredded dishcloth blue	Exfoliating gloves
Test 2	Dusters	Tissue paper balls	Yellow bath sponge
Test 3	Black satin	Cardboard	Rope
Test 4	Aspen	Cypress	Woodchip
Test 5	Purple ribbon	Green raffia ribbon	Sparkling fibre
Test 6	Cotton wool balls	Stringy cloth	Hair bands
Test 7	Fur	Polyester	Sparkly pompons
Test 8	Bin liner	Plastic scourer	Straws
Test 9	Brown pet bedding	Cork	Hessian sack
Test 10	Crepe paper squares	Scarf yarn	Pompons
Test 11	Coconut fibre	Coloured woodchip	Coloured matchsticks
Test 12	Absorbent fibre	String	Foam shapes
Test 13	Cellulose sponge	Corrugated paper	Perlite

### Training

On the first day of training, the animals were habituated to the arena. On the next days, the rats were trained to dig in a bowl filled with digging substrate (sawdust) to receive food reward (45-mg purified rodent tablets, TestDiet, Sandown Scientific, UK, catalogue #1811155, containing sucrose, casein, maltodextrin, corn starch, corn oil, minerals, silicon dioxide, vitamins, magnesium stearate, DL-methionine). Animals underwent three digging training steps. On the first day, each rat was placed in the test arena and given max. 3 min to approach and explore the empty (no substrate) bowl containing only two pellets. When the pellets were found and consumed, the trial was terminated, and the animal was removed from the arena and the bowl re-baited. During the next 2 days of training, each animal was given max. 30 s to explore and start digging for one pellet buried within 1 cm of sawdust. Following ten consecutive and successful digging trials in which the pellet was found and consumed, each rat was moved on to the next stage in which one pellet was buried within 2 cm of sawdust. Once each animal was able to find a pellet within 30 s on ten consecutive trials, the digging training was complete. On the last day of training, a discrimination session was presented allowing animals to explore two bowls with two novel digging substrates and a single buried pellet within one of them. Individual trials were run in which the animal was placed in the arena in front of the two bowls. Once the animal began to dig in one bowl, the other bowl was removed. Trials were continued with one substrate paired with the food pellet, until the rat attained six consecutive correct choices for that substrate.

### Pairing sessions and preference testing

**Fig. 1:**
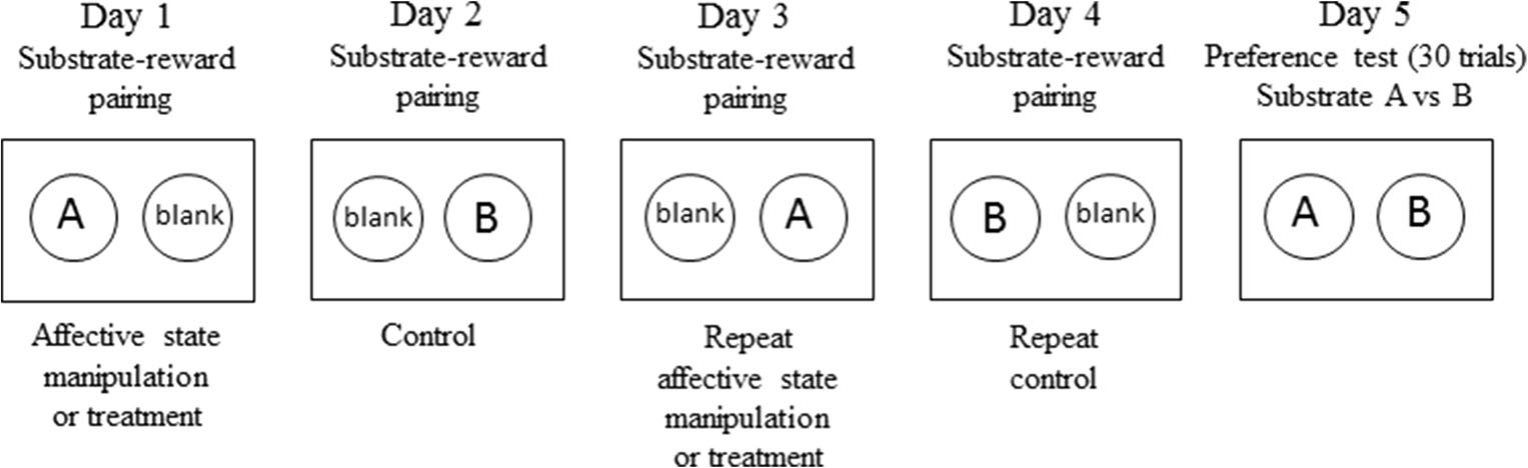
Method overview. Animals are subjected to four substrate–reward pairing sessions followed by the preference test. Adapted from Stuart et al. (2013)

Every study was based on four pairing sessions (4 days) followed by a preference test on the fifth day of that week (see Fig. 1). The rats encountered the two different substrate–reward pairings on separate days of the pairing sessions and were only presented with the two rewarded substrates together during the preference test. During pairing sessions, each animal learnt to associate two different digging substrates with obtaining a food reward under control or treatment conditions. The value of each experience was otherwise equal, and all factors (i.e. bowl location, substrates) were fully counterbalanced. Each trial involved choice between two bowls containing two different digging substrates: one ‘reward-paired’ (containing a food pellet) and the other a ‘blank’ substrate. The blank digging substrate was kept the same for the four pairing sessions, and a reward pellet was crushed into the bowl and mixed within the substrate, to avoid choices based on odour. Two reward-paired substrates were used: one presented on days 1 and 3 of the pairing sessions and the other on days 2 and 4. Presentation of one of these substrates (the ‘treatment-paired substrate’) was associated with administration of a treatment (e.g. drug administration) to the rat, whilst the other was not (see Fig. 1).

On each trial, the rat was individually placed in front of the two bowls and given 30 s to approach and choose the bowl. Once the rat made a choice and started digging in one bowl, the other bowl was removed. Choice of the reward-paired substrate was marked as a ‘correct’ trial, whilst digging in the blank substrate was classified as an ‘incorrect’ trial. An animal’s failure to approach and explore the bowls within 30 s was recorded as an ‘omission’. The number of trials and latency to dig were recorded for each animal. Once the rat achieved a criterion of six consecutive correct trials, the pairing session was completed.

Affective bias was measured during the preference test in which the two previously rewarded substrates were presented in bowls at the same time. Trials were reinforced using a random schedule with a single-pellet reward baited in either bowl with a probability of one in three. Both bowls also had a pellet crushed and placed in the substrate to reduce the likelihood of the animal using odour to find the reward. We also recorded the number of pellets each animal obtained during the choice trials and checked that the group average remained at chance (data not shown). This procedure was used to ensure that the animals remained motivated to approach the bowls and dig for reward, but they could not obtain new information about the substrate–reward association. This random reinforcement schedule also increased the probability of animals switching their choices between trials. This behaviour is thought to be an important factor in observing the affective bias. The animals’ choice and latency to dig were recorded. The number of choices made for the treatment-paired substrate versus the total number of trials was used as an index of bias.

### Drugs

Venlafaxine (3.0, 10.0 mg/kg, p.o., *t* = −30 min) was purchased from Tocris Bioscience, UK. Corticosterone (0.1, 1.0, 10.0 mg/kg, s.c., *t* = −30 min) and FG 7142 (3.0, 6.0 mg/kg, s.c., *t* = − 30 min) were purchased from Sigma-Aldrich, UK. Drugs were dissolved: venlafaxine in 1:1 distilled water and strawberry milkshake (Frijj, UK), corticosterone and FG 7142 in 5% DMSO and 95% sesame oil in a dose volume 1 ml/kg. Vehicle solution for venlafaxine was 1:1 distilled water and strawberry milkshake, whilst for FG 7142 and corticosterone, it was 5% DMSO and sesame oil. All drugs were used at doses based on previous studies (Stuart et al. 2013, 2015). Corticosterone was tested over a wider dose range as it had not previously been studied in the ABT in rodents. Prior to the start of the study, rats were trained to take milkshake (Frijj, UK; 0.5 ml) from a 1-ml syringe to facilitate oral drug dosing. On each day of treatment, drugs were freshly prepared and administered orally, i.e. venlafaxine, or subcutaneously, i.e. FG 7142 and corticosterone, 30 min prior to individual substrate–reward pairing sessions. For subcutaneous injections, animals were minimally restrained and injected on their left or right flank (alternating daily) to reduce the stress associated with restraint (Stuart and Robinson 2015a). Control animals received identical procedures but were injected with the vehicle only. All drug treatments were randomised using a fully counter-balanced study design and were performed blind to treatment.

### The effects of non-pharmacological manipulations: absolute value of reinforcer (one or two pellets), social play and psychosocial stress in male rats

To investigate the effects of the absolute value of reward, one substrate was paired with two reward pellets, whilst the other substrate was paired with a single reward pellet. On the fifth day, during preference testing, both digging substrates were equally reinforced with a single reward pellet.

Before beginning the social play manipulation, animals were habituated to an arena (100 × 100 × 50 cm) containing several toys (rope, platform, tennis balls, cardboard tubes, etc.) and free access to water for ~2 h per day for 1 week. Rats were habituated in the same social groups as would be used in the experiment with a total of six rats in the arena at the same time. For the ABT experiment, normal housing condition (control group) was paired with one substrate, whilst social play was paired with the other substrate. On the day that the animals were exposed to social play, the rats were placed in groups of six in the same highly enriched arena for a period of 8 h (9:00–17:00 h). During this time, each animal was removed individually and underwent one of the substrate–reward pairing sessions before being returned to the arena for the remainder of the enrichment period. All animals were returned to their home cage at 17:00 h. For the control condition, each animal underwent the substrate–reward pairing session whilst they were housed in their standard home cage.

Normal housing condition (control group) was paired with one substrate, whilst psychosocial stress was paired with the other substrate. To induce psychosocial stress, animals were exposed to restraint stress and social isolation. Rats from this group were subjected to 10 min in an immobilisation tube (restraint stress) and then underwent the substrate–reward pairing session immediately followed by ~8-h period of isolation housing. Isolated animals were kept in an unenriched cage with free access to water and paper partition between them to avoid visual contact. Rats were returned to their home cages at 17:00 h. For the control day, substrate–reward pairing sessions were carried out whilst rats were housed in their normal housing conditions.

### The effects of pharmacological manipulations with venlafaxine, FG 7142 and corticosterone in male rats

During pairing sessions, one substrate was paired with the drug (i.e. venlafaxine, FG 7142 or corticosterone) treatment whilst the other substrate was paired with the vehicle treatment. The absolute value of the reward (one pellet) was kept the same for each learning session.

### The effects of non-pharmacological and pharmacological manipulations: absolute value of reinforcer (one or two pellets), psychosocial stress, venlafaxine and corticosterone in female rats

Female rats were subjected to four experimental manipulations: high-value reward (one versus two pellets), restraint stress and psychosocial isolation and acute treatment with venlafaxine (3 mg/kg, i.p.) and corticosterone (10 mg/kg, s.c.). For pharmacological studies, the doses were chosen based on male rat results and all studies were carried out using the same methods as described above.

### Meta-analysis of effect sizes in the ABT in rats

We completed a quantitative analysis of the effect sizes of the pharmacological and non-pharmacological manipulations carried out in two different rat strains (SD, LH (data from Stuart et al. 2015; Stuart and Robinson 2015b) and both sexes in the ABT. Cohen’s *d* value was calculated to estimate the effect size, and the random effects model was used in the present meta-analysis. Cohen’s *d*, as a measure of the effect size, exhibits the difference between the treatment and control groups. Cohen’s *d* and the 95% confidence interval (CI) values were obtained for two study types: single manipulation studies (i.e. high-value reward, restraint stress and psychosocial isolation, social play) using equation for one sample *t* test and dose–response studies (i.e. venlafaxine, FG 7142, corticosterone) using formula calculating difference between the vehicle and the treatment dose. Cohen’s *d* estimator with 0.20, 0.50 and 0.80 values corresponded to small, moderate and large effects, respectively. The *I* ^2^ and *Χ* ^2^ statistics were used to explore the heterogeneity between studies. A statistical formula *I* ^2^ = *Q* − *dfQ* × 100%, where *Χ* ^2^ defines *Q* and *df* represents its degrees of freedom, was utilised to calculate *I* ^2^ (Huedo-Medina et al. 2006; Neyeloff et al. 2012). The percentage of *I* ^2^ indicates the level of heterogeneity: 0–25% not important, 25–50% small, 50–75% moderate and 75–100% high. A forest plot was created to visualise effect sizes between rat strains and sexes in the Microsoft Excel software (Fig. 5).

## Analysis

The sample size used in these studies was based on previous investigations and a power calculation (GPower3.1.9.2) using data reported in Stuart et al. (2013). Data were analysed using GraphPad Prism 6.0 (GraphPad Software, USA). Choice bias was calculated as the number of choices made for the treatment-paired substrate divided by the total number of trials (treatment-paired substrate + vehicle-paired substrate) multiplied by 100 to give a percentage value. A value of 50 was then subtracted to give a % choice bias score where a bias towards the treatment-paired substrate gave a positive value and a bias towards the control-paired substrate gave a negative value.

The % choice bias data from the dose–response studies were analysed using a repeated measures ANOVA with treatment as the within-subject factor. A Huynh–Feldt correction was used to adjust for violations of the sphericity assumption, Shapiro–Wilk test was used to determine a normal distribution, and Levene’s test was used to correct for inequality of variances for the % choice bias. Post hoc analysis for each drug dose and % choice bias data from single manipulation studies utilised a one-sample *t* test against a null hypothesised mean of 0% choice bias. For each animal, latency and trials to criterion during pairing sessions were recorded and analysed using a paired *t* test, comparing control/vehicle versus manipulation/treatment for the pairing sessions. Analysis of the choice latency and trials to criterion was made to determine the presence of any non-specific effects of treatment (i.e. sedation).

## Results

### Effects of absolute reinforcer magnitude on % choice bias

**Fig. 2:**
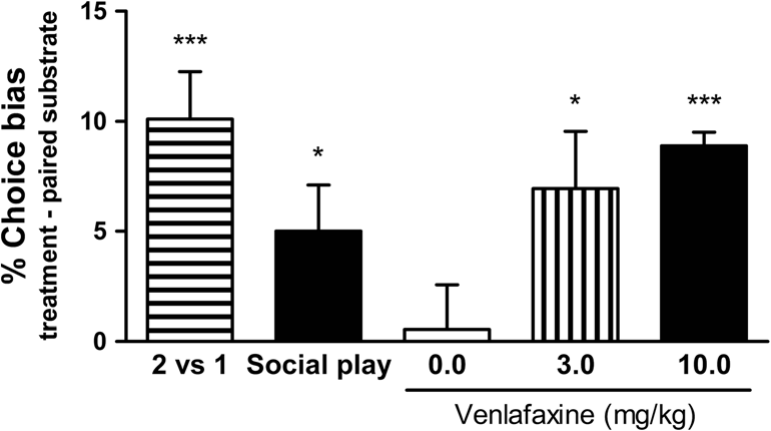
Substrates associated with psychosocial and pharmacological manipulations are preferred relative to those associated with control conditions, indicating induction of positive affective states by these manipulations in non-human animals. The two-pellet versus one-pellet pairing sessions show a positive bias towards the substrate previously associated with the higher value reward. The results illustrate induction of positive affective biases in rats following acute treatment with typical antidepressant, serotonin–noradrenaline reuptake inhibitor, venlafaxine. Rats also showed significant positive choice bias for substrates paired with social play. Data shown as mean % choice bias ± SEM; *p < 0.05; **p < 0.01; ***p < 0.001; one sample *t* test against a null hypothesised mean of 0% choice bias

**Fig. 3:**
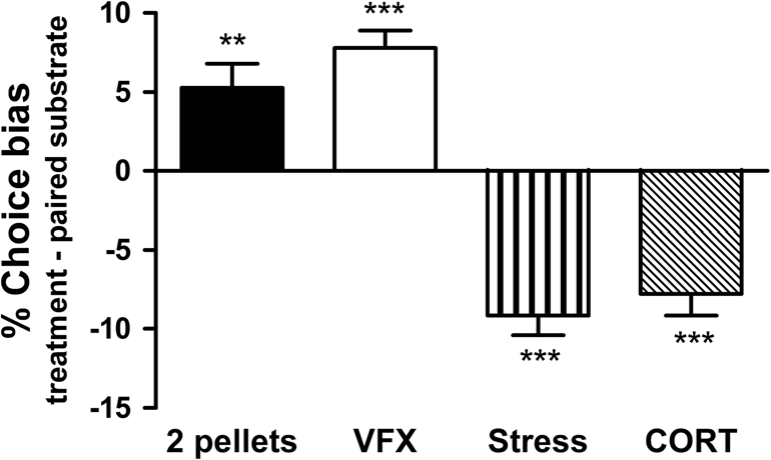
The substrate–reward pairing sessions using two pellets versus one pellet induced a positive affective bias towards the previously encountered high-rewarded substrate. Rats showed significant positive affective bias following acute treatment with typical antidepressant, serotonin–noradrenaline reuptake inhibitor, venlafaxine (VFX, 3 mg/kg). The results also demonstrate induction of negative affective biases in rats following acute corticosterone (CORT, 10 mg/kg) treatment as well as restraint stress and social isolation (RSSI) manipulation. Data shown as mean % choice bias ± SEM; *p < 0.05; **p < 0.01; ***p < 0.001; one sample *t* test against a null hypothesised mean of 0% choice bias

The substrate–reward pairing sessions using two reward pellets versus one reward pellet induced a subsequent positive affective bias towards the previously encountered high-rewarded substrate for both male (one sample *t* test, *t*_11_ = 4.716, *p* = 0.0006, Fig. 2) and female rats (one sample *t* test, *t*_11_ = 3.506, *p* = 0.0049, Fig 3). No differences were observed for latency or trials to criterion during the pairing sessions (Tables 2 and 3).

**Table 2:** Pairing session data following manipulations associated with a positive affective state

Experiment		Response latency (s)		Trials to criterion	
		Control	Manipulation	Control	Manipulation
2 Pellets (high-value reward)		2.9 ± 0.3	2.6 ± 0.2	10.7 ± 0.7	10.7 ± 0.7
Social play		1.7 ± 0.1	1.8 ± 0.1	7.4 ± 0.7	8.0 ± 0.4
Treatment	Dose (mg/kg)	Response latency (s)		Trials to criterion	
		Vehicle	Drug	Vehicle	Drug
Venlafaxine	0.0	1.8 ± 0.1	1.8 ± 0.1	6.7 ± 0.2	6.6 ± 0.2
	3.0	1.9 ± 0.2	2.0 ± 0.3	6.8 ± 0.2	6.5 ± 0.1
	10.0	1.8 ± 0.2	1.9 ± 0.2	6.5 ± 0.1	6.5 ± 0.1

Data shown as mean (*n* = 12 animals/group) ± SEM averaged from the two pairing sessions for each substrate–reward association (control/vehicle or manipulation/drug)

**Table 3:** Pairing session data following manipulations associated with positive and negative affective states in female rats

Experiment		Response latency (s)		Trials to criterion	
		Control	Manipulation	Control	Manipulation
2 Pellets (high-value reward)		2.9 ± 0.2	2.9 ± 0.2	8.2 ± 0.3	8.4 ± 0.3
Restraint stress and social isolation		2.2 ± 0.1	2.2 ± 0.2	6.5 ± 0.1	6.8 ± 6.02
Treatment	Dose (mg/kg)	Response latency (s)		Trials to criterion	
		Vehicle	Drug	Vehicle	Drug
Venlafaxine	3.0	2.0 ± 0.2	2.0 ± 0.2	7.2 ± 0.2	6.9 ± 0.1
Corticosterone	10.0	2.4 ± 0.2	2.2 ± 0.2	7.5 ± 0.2	7.6 ± 0.3

Data shown as mean (*n* = 12 animals/group) ± SEM averaged from the two pairing sessions for each substrate–reward association (control/vehicle or manipulation/drug)

### Effects of positive affective state manipulations

Acute administration of the mixed serotonin and noradrenaline reuptake inhibitor, venlafaxine, before the pairing session induced a dose-dependent positive bias towards the treatment-paired substrate for both male (3.0–10.0 mg/kg, RM ANOVA *F*_2.11_ = 4.305, *p* = 0.0264, Fig. 2) and female rats (3.0 mg/kg, one sample *t* test, *t*_11_ = 7.000, *p* = 0.0001, Fig. 3). Positive biases were observed for both 3.0 and 10.0 mg/kg doses (one sample *t* test, *p* < 0.05 and *p* < 0.001, respectively) in male rats. Only male rats underwent the social play manipulation and made significantly more choices for the substrate–reward association learnt during social play compared with the substrate–reward association learnt during control housing (one sample *t* test, *t*_11_ = 2.367, *p* = 0.0374, Fig. 2) indicating a positive affective bias. No significant differences during pairing sessions were observed for any treatment for either response latency or number of trials to criterion (Tables 2 and 3).

### Effects of psychosocial stress, FG 7142 and corticosterone on affective bias

**Fig. 4:**
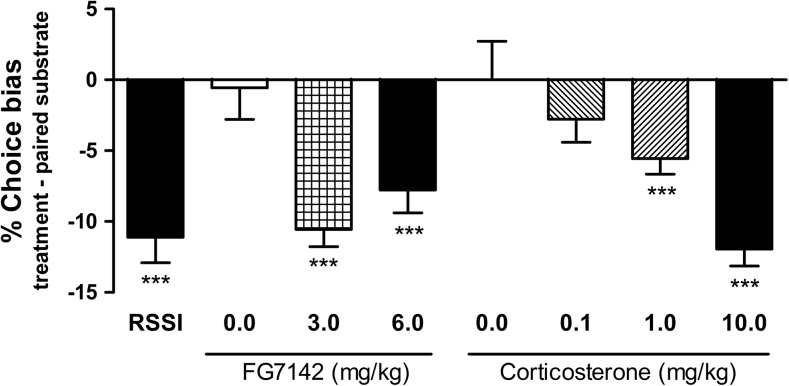
Acute treatment with stress-related hormone, corticosterone, anxiogenic benzodiazepine inverse agonist, FG 7142, and psychosocial stress are manipulations known to induce negative emotional states in non-human animals. Both substances, corticosterone and FG 7142, and non-pharmacological manipulation induced significant negative affective biases in rats following acute treatment. Data shown as mean % choice bias ± SEM; *p < 0.05; **p < 0.01; ***p < 0.001; one sample *t* test against a null hypothesised mean of 0% choice bias

Restraint stress followed by social isolation (RSSI) induced negative affective bias with animals making fewer choices for the substrate–reward association learnt during psychosocial stress compared with choices for the substrate–reward association learnt during normal housing for both male (one sample *t* test, *t*_11_ = 6.159, *p* = 0.0001, Fig. 4) and female rats (one sample *t* test, *t*_11_ = 7.396, *p* = 0.0001, Fig. 3).

Acute treatment with the stress hormone, corticosterone, induced a negative affective bias for both male (0.1–10.0 mg/kg, RM ANOVA, *F*_3.11_ = 7.224, *p* = 0.0007, Fig. 4) and female rats (10.0 mg/kg, one sample *t* test, *t*_11_ = 5.631, *p* = 0.0002, Fig. 3). Effects were observed for both 1.0 and 10.0 mg/kg doses (*p* < 0.001, one sample *t* test) in male rats.

Only male rats were treated with the anxiogenic benzodiazepine inverse agonist, FG 7142, and made fewer choices for the drug-paired substrate resulting in negative bias (3.0–6.0 mg/kg, RM ANOVA, *F*_2.11_ = 8.904, *p* = 0.0015, Fig. 4). Effects were observed for both 3.0 and 6.0 mg/kg doses (*p* < 0.001, one sample *t* test).

There were no significant effects during pairing sessions, either on response latency or number trials to criterion following treatment with corticosterone, FG 7142, or psychosocial manipulation (Tables 3 and 4).

**Table 4:** Pairing session data following manipulations associated with a negative affective state

Experiment		Response latency (s)		Trials to criterion	
		Control	Manipulation	Control	Manipulation
Restraint stress—social isolation		2.3 ± 0.2	2.2 ± 0.3	8.2 ± 0.5	8.7 ± 0.6
Treatment	Dose (mg/kg)	Response latency (s)		Trials to criterion	
		Vehicle	Drug	Vehicle	Drug
Corticosterone	0.0	1.4 ± 0.1	1.5 ± 0.1	6.4 ± 0.1	6.5 ± 0.1
	0.1	1.4 ± 0.1	1.5 ± 0.1	6.3 ± 0.1	6.3 ± 0.1
	1.0	1.5 ± 0.1	1.4 ± 0.0	6.4 ± 0.3	6.3 ± 0.1
	10.0	1.5 ± 0.1	1.4 ± 0.1	6.3 ± 0.1	6.5 ± 0.1
FG 7142	0.0	1.7 ± 0.1	1.5 ± 0.1	7.0 ± 0.2	7.0 ± 0.2
	3.0	1.6 ± 0.2	1.8 ± 0.3	6.7 ± 0.2	7.1 ± 0.4
	6.0	1.6 ± 0.1	1.7 ± 0.1	7.0 ± 0.2	7.0 ± 0.2

Data shown as mean (*n* = 12 animals/group) ± SEM averaged from the two pairing sessions for each substrate–reward association (control/vehicle or manipulation/drug)

### Meta-analysis of the effect size observed in male Lister Hooded versus male and female SD rats

**Fig. 5:**
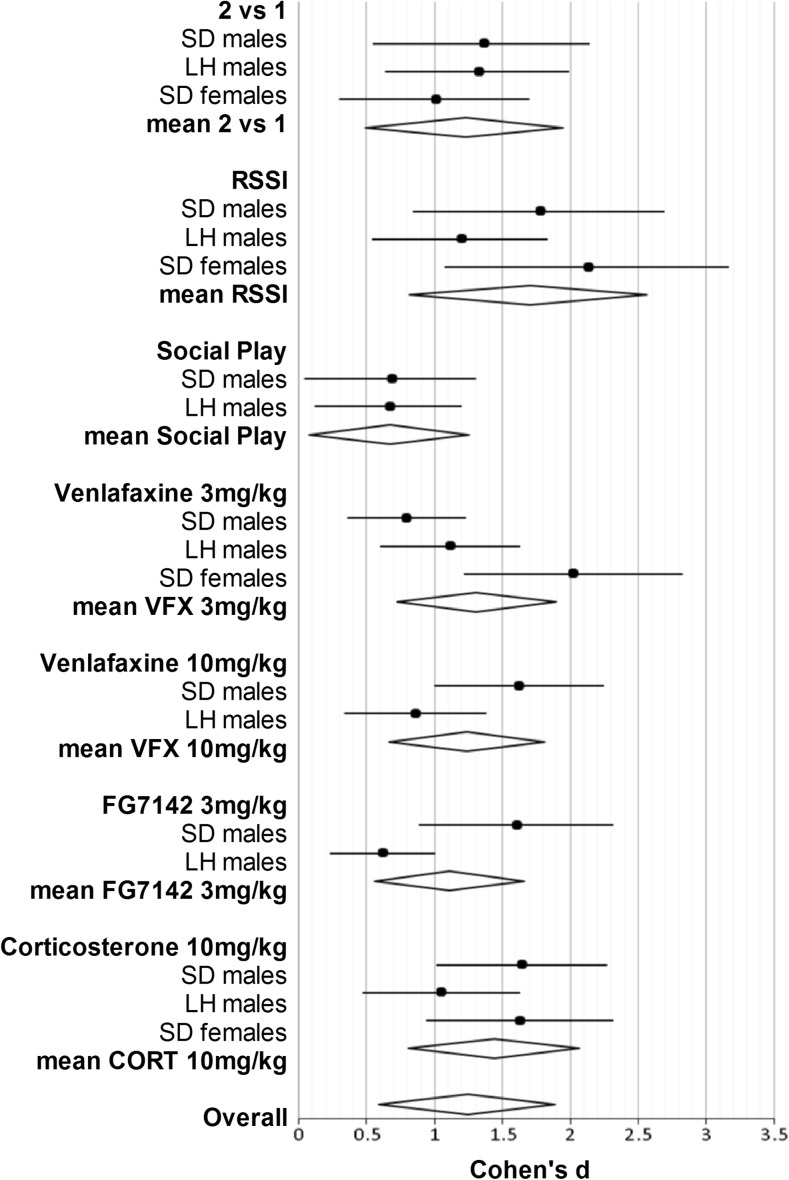
Meta-analysis of the effect size in the affective bias test. Forest plot showing the results of studies examining the pharmacological and non-pharmacological manipulations between males and females and two rat strains, Sprague Dawley (SD) and Lister Hooded (LH). The figure shows effect size estimator Cohen’s *d* for each study and the associated 95% confidence intervals

Overall, the results from the meta-analysis suggest that the ABT data for both pharmacological and non-pharmacological manipulations show moderate to large effect sizes. Similar results were observed for all three cohorts analysed with no evidence of heterogeneity between populations. Results are summarised in Fig. 5.

The three studies testing high-value reward manipulations (Cohen’s *d* = 1.36 (SD♂), 1.32 (LH♂), 1.01 (SD♀), Fig. 5), restraint stress and psychosocial isolation (Cohen’s *d* = 1.78 (SD♂), 1.20 (LH♂), 2.14 (SD♀), Fig. 5) showed a large effect size in all groups. A moderate effect size was found for social play experiment in both male groups (Cohen’s *d* = 0.68 (SD♂), 0.67 (LH♂), Fig. 5). Heterogeneity was not observed for high-value reward (*Q*(*df* 2) = 2.49, *I* ^2^ = 19.65%, *p* = 0.29), restraint stress and psychosocial isolation (*Q*(*df* 2) = 1.91, *I* ^2^ = 0%, *p* = 0.39) or social play (*Q*(*df* 1) = 1.00, *I* ^2^ = 0%, *p* = 0.32).

All pharmacological treatment studies yielded moderate or large effect size in both rat strains and sexes. The serotonin–noradrenergic reuptake inhibitor, venlafaxine, at 3 mg/kg had a moderate effect size for SD males (Cohen’s *d* = 0.79, Fig. 5), whilst LH males (Cohen’s *d* = 1.11, Fig. 5) and SD females (Cohen’s *d* = 2.02, Fig. 5) experiments exhibited large effect sizes. The higher venlafaxine dose of 10 mg/kg had a large (Cohen’s *d* = 1.62 (SD♂), Fig. 5) and moderate (Cohen’s *d* = 0.85 (LH♂), Fig. 5) effect size depending on the strain tested. Studies with use of pro-depressant drug, FG 7142, showed large and moderate effect sizes for SD males (Cohen’s *d* = 1.60, Fig. 5) and LH males (Cohen’s *d* = 0.62, Fig. 5), respectively. In both rat strains and both sexes, corticosterone treatment resulted in a large effect size (Cohen’s *d* = 1.64 (SD♂), 1.05 (LH♂), 1.63 (SD♀), Fig. 5). The heterogeneity tests for venlafaxine study, 3 mg/kg (*Q*(*df* 2) = 2.50, *I* ^2^ = 20.03%, *p* = 0.29) and 10 mg/kg (*Q*(*df* 1) = 1.00, *I* ^2^ = 0%, *p* = 0.32), were not statistically significant. In addition, no significant heterogeneity was indicated in FG 7142 3 mg/kg study (*Q*(*df* 1) = 1.00, *I* ^2^ = 0%, *p* = 0.32) and corticosterone 10 mg/kg study (*Q*(*df* 2) = 1.98, *I* ^2^ = 0%, *p* = 0.32).

## Discussion

Undertaking a series of experiments in both male and female SD rats, we have been able to show that affective biases, measured using the ABT, provides a robust, predictive model for antidepressant research. The results for the antidepressant and pro-depressant drug treatments were consistent with the effects seen in Lister Hooded male rats. We also add to this validation by showing that acute treatment with the stress hormone corticosterone induces a negative affective bias in both sexes. Stress is a major risk factor for the development of depression, and previous work in rodents has shown that chronic stress or corticosterone treatment induces a putative model of depression (Blanchard et al. 2001; Marais et al. 2008; Murray et al. 2008). However, being able to link the acute effects of corticosterone to a negative affective bias is an important advance. This finding suggests that acute increases in corticosterone induce similar effects to other pro-depressant treatments in this test and concurs with our hypothesis that biases in the ABT are predictive of long-term pro-depressant risk. Using a meta-analysis, we also compared data for both rats strains and the different pharmacological and psychosocial manipulations and overall found robust effect sizes with similar results seen across the different cohorts. The following discussion considers these effects in more detail and also the wider implications of the work in relation to neuropsychological mechanisms and their role in the cause and treatment of major depression.

Corticosterone is the stress hormone suggested to play a causal role in the development of depression. Evidence from animal studies supports this hypothesis as exposure of either rats or mice to chronic corticosterone treatment induces depression-like behaviours in conventional models of depression, such as the forced swim test and sucrose preference test, and neurotrophic changes in the brain (Ambrogini et al. 2002; Ardayfio and Kim 2006; Brummelte and Galea 2010; David et al. 2009; Rygula et al. 2005). In our ABT, corticosterone induced a dose-dependent negative bias in both the male and female rats tested and at doses which had no effect on learning or motivation during the pairing sessions. The effects of corticosterone treatment are similar to those seen with restraint stress and social isolation both in this study and in our previous publications (Stuart et al. 2013, 2015; Stuart and Robinson 2015b). The acute effects of corticosterone also concur with previous work published for a different affective bias task, the judgement bias test or ambiguous cue interpretation task (Enkel et al. 2010). In this task, Enkel et al. (2010) showed that treatment with acute corticosterone in combination with a noradrenaline re-uptake inhibitor induced a negative bias with animals more likely to make a pessimistic choice following acute treatment. These findings suggest this stress hormone has an acute, neuropsychological effect which, we propose, may be important for its long-term effects on mood in man (Gold et al. 2015; Hardeveld et al. 2014; Vrieze et al. 2015). Chronic stress and stressful life events are major risk factors for developing major depression (Beck 2008; Clark et al. 2009), and high plasma corticosterone and corticotrophin-releasing factor in cerebrospinal fluid have been observed in depressed patients (Juruena et al. 2004; Merali et al. 2004). These observations have led to the hypothesis that increased levels of stress hormones over a sustained period could trigger a depressive episode (Beck 2008).

Negative affective biases were also observed in male and female SD rats following a psychosocial stress involving restraint stress and social isolation and in males following acute treatment with the anxiogenic drug, FG 7142. Similar biases were observed in both sexes, and the overall effects were consistent with previous studies and also of a similar magnitude to the effects of corticosterone. The meta-analysis data show that moderate to large effect sizes were seen following these different treatments. When these negative bias data are considered together, it is interesting to observe that all three treatments have been linked with an increase in corticosterone level. FG 7142 has been shown to activate the HPA and stimulates corticosterone release (Mikkelsen et al. 2005). Stressors, especially psychosocial stressors, elevate endogenous corticosterone levels and are widely used to induce negative affective states and model depression in animals (Nestler and Hyman 2010; Papp 2012; Pollak et al. 2010; Willner 1984). Our findings suggest that exposure to treatments that lead to elevations in glucocorticoids induces negatively valenced cognitive processing that results in a subsequent negative bias for an experience encountered during the treatment (Beck 2008; Disner et al. 2011; Drevets 2001).

The results for manipulations inducing biases in a positive direction also found similar results for both male and female SD rats. The meta-analysis data confirm that the treatments investigated had moderate to large effect sizes, and similar data were obtained for the different sexes and strains tested to date. The experiment using the higher value reward provides proof-of-concept data and demonstrates that rats will bias their responding based on their previous experience of the different value of reward associated with each digging substrate. It also provides a useful benchmark in terms of the level of bias which a change in absolute reward will induce relative to the effects seen using affective state manipulations or drug treatments and a reward value which is kept constant. Positive biases were also observed for social play and acute treatment with the antidepressant, venlafaxine, consistent with our previous findings in the Lister Hooded rat (Stuart et al. 2013). Here, the absolute value of the reward during learning is the same and the bias is therefore attributed to the effect of the treatment.

Antidepressant drug treatments have also been shown to induce positive biases in human (Harmer et al. 2009b, 2004) and non-human animals (Anderson et al. 2013; Rygula et al. 2014) following acute treatments. In this study, the effects of venlafaxine were dose-dependent and similar to previous observations in Lister Hooded rats (Stuart et al. 2015). Socially enriched environments have been shown to induce positive emotional processing in humans and non-human animals (see review of Mendl et al. 2009), as well as promoting synaptogenesis (Briones et al. 2004; Diamond et al. 1964, 1966), increasing dendrite complexity (Faherty et al. 2003; Leggio et al. 2005) and reducing heart rate and corticosterone levels (Kemppinen et al. 2010; Laws et al. 2007). It was interesting to observe that the effect size for social play was smaller than for some of the other manipulations. It is not clear whether this relates to a weaker overall effect in terms of its impact on affective state or that the social-enriched environment may not provide a positive environment for all individuals. Because social order may affect the stress levels of individuals, animals of lower social ranking may find this experience more stressful, hence resulting in higher variance in the data. Studies combining measures of social hierarchy and results from the ABT would be an interesting way to investigate this further.

The animals showed similar patterns of learning under drug or vehicle treatment during the pairing sessions. There was also no effect on latency during the pairing sessions for any of the treatments tested. This suggests that choice bias was not related to enhancement or impairment in learning or changes in motivational state induced by the drug treatments. This is consistent with our proposal that the observed biases in this task represent a specific effect on how the value of experience is learnt and the impact that this has on subsequent behaviour. We suggest that the affective state at the time of learning can modify the overall valuation of the rewarding experience such that it is relatively enhanced or reduced leading to a bias in behaviour when the reward-paired cues (digging substrates) are re-encountered.

The results obtained for the male versus female rats suggest that similar biases are observed in each sex although the cohorts were not run in parallel, and hence, a direct statistical comparison was not possible. The results from the meta-analysis did show similar effect sizes for the different manipulations, both pharmacological and psychosocial, in both sexes. Evidence from population studies in humans suggests that females are more susceptible to mood disorders with a much higher prevalence of patients being female (Bekhbat and Neigh 2017; Kessler 2003; Swaab et al. 2005). The fact that the ABT works well in both sexes offers the possibility of using the task in future studies to investigate sex differences.

Alongside work with the JBT (for reviews, see Hales et al. 2014; Mendl et al. 2009), there is increasing evidence from animals that affective biases relevant to MDD can be quantified using non-human species. This means that more specific and well-controlled translational experiments can be undertaken to investigate hypotheses relating to neuropsychological mechanisms in depression (Disner et al. 2011; Harmer et al. 2009a; Roiser et al. 2012). It also offers new methods of measuring animal affect that have relevance in neuroscience, psychopharmacology and animal welfare science.

Animals provide a valuable tool for identifying novel drug targets as well as testing potential efficacy of novel treatments; however, for MDD research, conventional animal tests such as the forced swim test and tail suspension test have been heavily criticised (Pollak et al. 2010; Slattery and Cryan 2012; Willner 1984). It is hoped that quantification of affective biases might offer an alternative method to predict the pharmacological effects of novel antidepressants and assess pro-depressant risk (Stuart et al. 2013, 2014). This work also adds to the wider discussion relating to the role of neuropsychological mechanisms in the cause and treatment of major depression (Clark et al. 2009; Disner et al. 2011; Roiser et al. 2012). Studies with antidepressants now suggest that they have acute biochemical effects which can be seen in patients and healthy volunteers when looking at objective measures of emotional processing (Harmer et al. 2017, 2009a, 2004; Pringle and Harmer 2015). This work underpins the hypothesis that the delayed onset of action of antidepressants may be related to these effects and the time needed for these objective effects to change the patients’ experience of their environment such that subjective changes in mood can develop. Our findings with acute corticosterone reveal that this important risk factor for depression can induce negative biases opposite to those seen with antidepressants but similar to those previously observed for other pro-depressant treatments, e.g. the CB1 antagonist, rimonabant (Stuart et al. 2013).
